# Strategic integration of 3D Cell culture and regenerative biomedical technologies in India’s biomedical ecosystem: Aligning with the global transition toward human-relevant drug development

**DOI:** 10.1016/j.namjnl.2026.100116

**Published:** 2026-07-16

**Authors:** Prajakta Dandekar, Ratnesh Jain, Surat Parvatam, Viraj Mehta

**Affiliations:** a Department of Pharmaceutical Sciences and Technology, Institute of Chemical Technology, Mumbai, India; b Department of Biological Sciences and Biotechnology, Institute of Chemical Technology, Mumbai, India; c Humane World for Animals India, Hyderabad, India; d Sai Life Sciences, Hyderabad, India

**Keywords:** New approach methods, Microphysiological systems, Animal alternatives, 3d cell culture

## Abstract

Three-dimensional (3D) cell-culture and organ-mimetic systems are transforming biomedical research by offering physiologically relevant models for drug screening, toxicology and regenerative medicine. With enactment of the U.S. FDA Modernization Act 2.0 (2022) and the introduction of Act 3.0 (2024), the European Medicines Agency (EMA)’s endorsement of new approach methodologies (NAMs) and UK government’s roadmap to replace animal testing (2025) the regulatory science has shifted toward human-relevant models. However, the development of these technologies in India remains limited to a few leading institutes. This manuscript presents a perspective on adoption of a national strategy for popularizing 3D culture and regenerative biomedical solutions within Indian institutions, with the long-term goal of integrating them into the central drugs standard control organisation (CDSCO), biotechnology for economy, environment, and employment (BioE3) and anusandhan national research foundation (ANRF) mandates, thus aligning them with the India Vision 2047. It reviews Indian and global landscape, identifies skill and infrastructure gaps, and proposes policy and implementation frameworks, emphasizing on capacity building, regulatory incentives and industry-academia collaboration. A roadmap for establishing regional hubs and harmonized standards has been proposed to enable India to emerge as a Global South leader in ethical and cost-effective alternatives-to-animal testing.

## Highlights

• Proposes a national NAMs roadmap for India’s biomedical ecosystem.
• Aligns 3D culture adoption with national initiatives, India Vision 2047 and BioE3.
• Recommends regional hubs for organoid and organ-on-chip research.
• Provides policy and institutional framework to link policy, funding and industry.
• Provides recommendation to develop 3D cell culture ecosystem in India.

## 1 Introduction

### 1.1 Rationale and context

The biomedical sector is witnessing a paradigm shift toward human-relevant, in vitro models that can complement or replace animal testing. Conventional two-dimensional (2D) cell culture systems have served as indispensable tools in biomedical research for decades. However, they fail to replicate the three-dimensional architecture, extracellular matrix composition, mechanical stimuli, oxygen and nutrient gradients, and complex cell-cell interactions that regulate tissue function in vivo. Three-dimensional culture systems, including spheroids and organoids, overcome many of these limitations by recreating key aspects of the native tissue microenvironment, thereby enabling more physiologically relevant cellular responses (Bell et al., 2018; Zhao et al., 2022). Building upon these advances, microphysiological systems (MPS) integrate engineered microfluidics with human cells to reproduce tissue-level architecture, vascular perfusion, biomechanical forces, and inter-organ communication (Ingber, 2022). Compared with traditional animal models, MPS minimize uncertainties arising from species-specific differences in drug metabolism, immune responses, and disease biology, resulting in improved prediction of human pharmacokinetics, pharmacodynamics, efficacy, and toxicity (Mehta et al., 2024; Keuper-Navis et al., 2023)

3D cell-culture encompasses various technologies such as spheroids, organoids, 3D bioprinting, hydrogel embedded cultures, and organ-on-chip (OOC) platforms that recapitulate tissue architecture and microphysiological cues far better than the conventional 2D cell monolayers (Ingber, 2022; Zhao et al., 2022; Baptista et al., 2022). While most microphysiological systems (MPS) consist of 3D biological structures, not all 3D cell culture systems qualify as MPS because they may lack perfusion, biomechanical stimulation, or multi-tissue and multi cellular interactions. While we have discussed 3D culture technologies within the broader NAM ecosystem in this paper; particular emphasis has been placed on MPS and advanced organoid-based platforms because of their growing importance in human-relevant drug development, disease modelling, and regulatory decision-making.

It is important to recognize that not all three-dimensional culture systems provide equivalent physiological complexity. Conventional spheroids and organoids cultured in static multi-well plates represent an important advance over two-dimensional cultures by recapitulating tissue architecture, cell-cell interactions, cellular heterogeneity, and extracellular matrix interactions (Wang et al., 2025). However, these systems generally lack vascular perfusion, physiologically relevant mechanical forces, and dynamic control over the cellular microenvironment. Consequently, their ability to model systemic physiology, long-term tissue function, and drug pharmacokinetics remains limited (Keuper-Navis et al., 2023).

MPS particularly organ-on-chip technologies, address many of these limitations by integrating engineered microfluidics with human tissues to recreate continuous perfusion, tissue-tissue interfaces, mechanical stimuli, and real-time functional monitoring. Representative examples include the Wyss Institute's lung-on-a-chip, which reproduces the alveolar-capillary interface under cyclic mechanical stretching to mimic breathing (Huh et al., 2010). The next generation of MPS comprises interconnected multi-organ platforms, exemplified by Hesperos' multi organ platform, which links multiple human organ models through a common recirculating microfluidic system to investigate organ-organ interactions, systemic pharmacokinetics, pharmacodynamics, and off-target toxicities (McAleer et al., 2019; McAleer et al., 2019). These increasingly sophisticated platforms bridge the gap between conventional in vitro assays and clinical physiology, thereby representing the current state-of-the-art in human-relevant drug development and NAMs.

A significant point of deliberation is that although 3D culture systems and MPS provide enhanced physiological relevance compared with conventional monolayer cultures, they may increase assay complexities due to various reasons, such as variability in cell sources, non specific binding, shear forces due to fluid flow, and media compositions for different cell types [12]. Hence, model selection should be guided by the intended Context of Use (CoU) or the specific regulatory and scientific context for the model has been designed. For high-throughput screening, conventional 2D systems may often be more appropriate due to their simplicity, scalability, and robustness. In contrast, MPS may become particularly valuable when tissue architecture, multicellular interactions, organ-organ crosstalk, biomechanical forces, pharmacokinetic behaviour, or human-specific responses are critical for determining the biological outcome or making mechanistic decisions. In particular human relevant microphysiological systems are a reliable option for the safety and efficacy testing of new modalities and biologics for which relevant animal species are not available.

This physiological relevance has now been acknowledged and enforced through the global regulatory reforms, like the U.S. FDA Modernization Act 2.0 enacted in December 2022, which has removed mandatory animal trials to ascertain safety and efficacy of drugs and the Modernization Act 3.0, introduced in February 2024, that has directed the FDA to define validation pathways for alternative models, such as organ-on-chips and computational models etc. [(S.5002 - 117th Congress (2021–2022): FDA Modernization Act 2.0, 2026; FDA Modernization Act 2.0, FDA Modernization Act 3.0)]. In Europe, the shift towards new approach methodologies (NAMs) is anchored in a formal regulatory framework. At the legislation level, Directive 2010/63/EU on the protection of animals used for scientific purposes has set the long-term goal of phasing out the use of animals and requires that validated alternatives be used whenever available, a principle that is reinforced across chemicals legislation and Regulation on the registration, evaluation, authorisation and restriction of chemicals (REACH), where animal tests are permitted only as a last resort. Building on this, the European Medicines Agency (EMA) has issued specific web-based guidance on “Regulatory acceptance of new approach methodologies (NAMs) to reduce animal use in testing”, which explains when and how NAMs data (including in vitro models and microphysiological systems) can support non-clinical development and how sponsors should seek scientific advice or Innovation Task Force (ITF) briefing meetings to discuss these approaches. In parallel, the EU Reference Laboratory for Alternatives to Animal Testing (EURL ECVAM) coordinates formal validation studies, issues scientific recommendations, and feeds successful methods into the organisation for economic co-operation and development (OECD) Test Guideline programme. For example, TG 492 on reconstructed human cornea-like epithelium for eye irritation and TG 497 on defined approaches for skin sensitisation, thereby turning NAMs into internationally recognised regulatory tools that are directly applied by EU regulators [(European Union, 2010; Marinou and Dontas, 2023; European Commission, 2026.; OECD, 2025; OECD, 2025)]. Recent EU “horizon scanning” and roadmap documents further commit to progressively transforming chemical and safety legislation so that animal testing is phased out wherever scientifically feasible, with NAMs as the default option [(European Medicines Agency EMA, 2026; Uster et al., 2024; New Approach Methodologies EU-IN Horizon Scanning Report, 2026)]. The recently proposed EU Biotech Act explicitly recognized the potential of NAMs for accelerating the drug discovery and proposed *health biotechnology strategic projects* recognition for the projects utilizing NAMs (European commission, 2025.). Recently announced UK government’s policy paper is one of the most detailed strategic plan to replace animal testing. Following this announcement, medicines and healthcare products regulatory agency (MHRA) has published a guidance document outlining specific cases where NAMs usage is warranted (MHRA, 2026; Government of UK, 2025). Health Canada (HC) and Environment and Climate Change Canada (ECCC) have also released a strategy document outlining the importance of replacing the use of vertebrate toxicity tests with non-animal methods (Government of Canada, 2025). Together, these developments suggest a future where 3D cell culture and microphysiological systems (MPS) will be essential tools in drug development.

In India, these emerging human-relevant systems could have significant impact for drug development in areas of significant disease burden in India, including non-communicable diseases, such as cardiovascular diseases and diabetes; infectious and vector-borne diseases, such as Tuberculosis, Malaria etc. Apart from drug development, the use of 3D cell culture could play a significant role in vaccine safety evaluation, biosimilar functional characterization, cell and gene therapy potency testing as these sectors also represent significant market size in India. Also, India being one of world's largest agrochemical producers and exporters, MPS provides a significant opportunity support human-relevant toxicity assessment and mechanistic evaluation of in that sector as well.

India cannot sustain affordable biotherapies like biosimilars, complex biologics, mRNA vaccines, CAR-T, and regenerative therapeutics etc. without shifting to human-relevant 3D culture and NAM platforms. Animal models are increasingly inadequate, expensive, and globally being phased out, while the 2D assays lack predictive power. If India does not adopt validated 3D/NAMs systems at scale, the country will lose its cost advantage, face higher development failures, and risk long-term dependency on foreign technologies. Institutionalising these platforms is the only viable path to economical and globally competitive biodrug development. While the adoption of 3D culture and organ-mimetic technologies is expanding across multiple institutional ecosystems, it remains largely fragmented. A growing number of centres, including the biotechnology research and innovation council (BRIC) network institutions, Indian institute of technology (IITs), Indian institute of science (IISc), Indian institutes of science education and research (IISERs), council of scientific and industrial research (CSIR) laboratories, all India institute of medical sciences (AIIMS) hospitals, and translational hubs such as Institute of Chemical Technology, Mumbai, India (ICT, Mumbai) etc., are actively engaged in research pertaining to organoids, organ-on-chips and 3D bioprinting. Despite this encouraging landscape, these initiatives are often undertaken in silos, without a unified national framework for standardization, resource sharing or regulatory alignment. Thus, establishing a coordinated policy and funding mechanisms to link these efforts under a common translational roadmap is critical to accelerate India’s leadership in this emerging domain. Such a strategy will align with the goals of India’s Vision 2047, namely self-reliance and ethical innovation. It will also coordinate with the biotechnology for economy, environment, and employment (BioE3) policy for enabling biomanufacturing excellence and the Anusandhan national research foundation (ANRF) Centre of Excellence scheme for promoting translational biomedical R&D (Department of Biotechnology, 2026; BIRAC, 2024; ANRF, 2026).

### 1.2 Evolution of 3D cell-culture technologies

3D culture has evolved from simple multicellular spheroids to organoids derived from stem cells/patient derived cells and, more recently, to microfluidic organ-on-chips platforms. These models permit inter organ connection, continuous perfusion, longer culture times, gradient control, and real-time monitoring of cellular functions, and hence provide superior predictivity of pharmacokinetics and toxicity parameters (Keuper-Navis et al., 2023). Bioreactors now enable reproducible large-scale production of 3D models suitable for automation and high-throughput screening (Licata et al., 2023). Emerging integrations with AI and machine learning allow automated image analyses, simulation of disease phenotypes, and predictive modelling (Nosrati and Nosrati, 2023; Wang et al., 2024). While the scientific advantages are clear, translation into institutional practice depends on policy support, infrastructure investment, and workforce training, areas where India face substantial gaps. This manuscript articulates a strategic approach for embedding 3D cell culture and regenerative biomedical technologies as foundational components of India’s biomedical ecosystem.

## 2 Global and indian landscape

### 2.1 Global models and regulatory evolution

Global leadership in 3D cell culture integration stems from multi-agency consortia that link academia, regulators and industry. The NIH Tissue Chip Program (USA) has invested over US $150 million since 2012 to create human organ-mimetic systems validated in partnership with FDA and Defence Advanced Research Projects Agency (DARPA) (NCATS, 2026) The Harvard Wyss Institute and Fraunhofer Gesellschaft at Germany demonstrated how technology transfer from academia to biotech startups can accelerate commercialization (Wyss Institute, 2026; Fraunhofer Institute for Ceramic Technologies and Systems IKTS, 2026). The BOKU Centre for Biotechnology in Austria and the Bioengineering Centre at the National University of Singapore (NUS) offer models of interdisciplinary hubs where biology, materials science, and engineering converge. Japan’s Agency for Medical Research and Development (AMED)-funded organoid programs have established national biobanks and stem-cell repositories supporting clinical translation (Ishida, 2021).

The commercial MPS ecosystem encompasses not only technology developers but also specialized contract research organizations (CROs) and service providers that enable broader industrial adoption of human-relevant models. While product-based companies, such as Emulate Inc. primarily develop organ-on-chip platforms for research and regulatory applications; service-based MPS companies, including Hesperos Inc. and Dynamic42 GmbH provide contract research services utilizing multi-organ and disease-specific microphysiological systems for drug efficacy, pharmacokinetic, and safety evaluation. Hesperos' multi organ platform has been applied to investigate pharmacology, efficacy, and toxicity by integrating interconnected human tissue models, whereas Dynamic42 offers customized organ-on-chip services for disease modelling, infection modeling, toxicology, and PK applications. Similarly, Hub Organoids provides organoid drug screening services and custom organoid development services. The emergence of such service-oriented companies demonstrates that the MPS ecosystem has evolved beyond hardware development toward an integrated translational infrastructure that combines platform innovation with preclinical testing services. Similar business models could serve as valuable examples for developing India's future NAMs-enabled CRO ecosystem through collaborations between academic institutions, technology developers, and pharmaceutical companies.

There are also industry consortiums, such as the IQ-MPS, that provides a venue for appropriate cross-pharma collaboration, data sharing and publishes manuscripts containing considerations for developing, evaluating, and characterizing MPS models of various organs. Similarly, multi-stakeholder forums, such as European organ on chip society (EUROoCS), the Industry Alliance for Microphysiological Systems (IAMPS) and International MPS society (iMPSS) can bring together academic, industry, government communities, and provide a global platform for scientific exchange, community building and international collaboration, leading to accelerated innovation and adoption of MPS technologies.

International advocacy organizations, such as Humane World for Animals (previously Humane Society Internation) and Cruelty Free International (CFI) work globally with governments, regulators, and scientific communities to promote the implementation of NAMs through policy engagement, scientific capacity building, and regulatory advocacy aimed at reducing animal use in research and safety assessment. The International Foundation for Ethical Research (IFER) has provided sustained research funding supporting the development, validation, and adoption of innovative non-animal methodologies. Collectively, these organizations complement governmental initiatives by creating an international ecosystem that integrates scientific innovation, regulatory science, industry participation, education, and public policy, thereby accelerating the global transition toward predictive, human-relevant biomedical research.

The evolution of regulatory frameworks is occurring at a parallel pace. The FDA’s Alternative Methods Working Group established in 2023 now recognizes MPS data for investigational new drug (IND) submissions (FDA, 2025), while the EMA’s Innovation Task Force has issued procedural guidance for including organoid data in pre-submission meetings (European Medicines Agency EMA, 2026). List of Organisation for economic co-operation and development (OECD) guidelines that validated NAMs for safety assessment is mentioned in Table S1. These precedents define the pathway for Central Drugs Standard Control Organisation (CDSCO) to implement similar validation standards for Indian laboratories.

### 2.2 Indian initiatives and emerging hubs

India’s participation in 3D cell culture research is growing, though still modest compared with its western counterparts. Center for Predictive Human Model Systems (CPHMS) has recently published an interactive database of Indian MPS researchers highlighting growing NAMs ecosystem (AIC-CCMB, 2026). Collaborative centres, like the Mumbai Biocluster and Centre for High Precision Manufacturing Sciences, CPHMS, serve as translational interfaces between academia and industry. Recent biotechnology industry research assistance council (BIRAC) schemes, BioE3, biotechnology ignition grant (BIG), and small business innovation research initiative (SBIRI), have initiated supporting startups developing 3D culture reagents and microfluidic systems (Department of Biotechnology, 2026; BIRAC, 2024). Besides, Indian council for medical research (ICMR) has explicitly recognized the importance of non-animal models in drug development in their guidelines for funding. As an immediate action plan, in 2025, ICMR dedicated 5% of their *Small Extramural Grant* for supporting projects related to NAMs.

Nevertheless, India lacks an overarching regulatory and funding mechanism to harmonize these efforts. Unlike FDA and EMA, CDSCO does not yet have formal guidance on validating or accepting data from 3D or organ-on-chip models. Establishing a National Task Force on Alternative Methods under CDSCO could catalyse standardization, drawing from the experience of OECD and U.S. Interagency Coordinating Committee on the Validation of Alternative Methods (ICCVAM). Table 1 presents a comparative summary of the global and Indian 3D culture hubs and regulatory frameworks.

**Table 1:** Comparative summary of global and Indian 3D culture hubs and regulatory frameworks.

**Region / Country**	**Examples of leading MPS centers or related Programs/initiatives**	**Key Focus Areas**	**Regulatory / Policy Frameworks**	**Representative Achievements / Learnings for India**
United States	NIH Tissue Chip Program (NCATS); Complement ARIE program; Translational Centres for Microphysiological Systems	Organ-on-chip, microphysiological models, disease modelling, drug toxicity, regulatory science	FDA Modernization Acts 2.0 (2022) and 3.0 (2024); FDA Alternative Methods Program; FDA ISTAND program; FDA roadmap to reducing animal testing in preclinical safety studies	Formal regulatory acceptance of 3D and MPS data for IND filings; validated models for liver, kidney, and lung toxicity testing; Formal qualification program for innovative DDTs
Europe (EU)	EU EURL-ECVAM Network; Horizon Europe; Various 3R centres	Standardization, toxicology, biomaterials, GMP-grade manufacturing	EMA Innovation Task Force; EURL ECVAM; OECD NAMs guidelines for chemical testing (skin toxicity, ocular toxicity)	Harmonized validation frameworks for reconstructed human tissues; inter-laboratory reproducibility protocols.
United Kingdom	NC3Rs (UK Research and Innovation Council)	3Rs (Replace, Reduce, Refine) alternatives; MPS validation	UK Home Office Animals (Scientific Procedures) Act; NC3Rs funding mandate; UK roadmap to replace animal testing (2025)	Direct linkage between research funding and animal use reduction; policy-driven NAM adoption; Strategy to Prioritize replacement of animal tests
Japan	AMED Regenerative Medicine Program (AMED-MPS);	Organoid biobanks, stem-cell derivation, disease modeling	Pharmaceuticals and Medical Devices Agency (PMDA)guidelines for cell-based products (2023); Ethical Guidelines for Human Stem Cells	Integration of stem-cell policy with national biobanking initiatives.
India	Indian council of medical research (ICMR) extramural grants; Biotechnology Industry Research Assistance Council (BIRAC) - Establishing pre clinical models for drug discovery	3D bioprinting, organoid development, bioprocess scale-up, nanomedicine	Amendment to New Drugs and Clinical Trials rules (2023); Evolving CDSCO framework; DBT–BIRAC BioE3 Mission; ICMR Stem-Cell Guidelines (2023)	Emerging centers of excellence; need for CDSCO validation pathway and national coordination through BioE3 and ANRF programs.

## 3 Challenges in 3D culture adoption in india

**Fig. 1:**
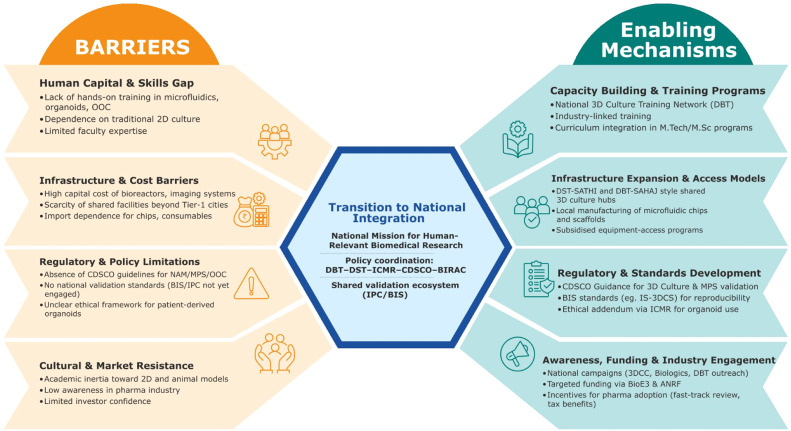
Schematic of key barriers and enabling mechanisms for 3D culture adoption in India.

Despite promising beginnings, India faces multi-dimensional barriers to the widespread adoption of 3D culture and regenerative biomedical platforms. These can be grouped into four macro-themes: (a) human capital and training, (b) infrastructure and cost, (c) regulatory and policy limitations, and (d) cultural and market barriers, as depicted in Fig. 1.

### 3.1 Human capital and skill gaps

Development of 3D microphysiological systems demands multidisciplinary expertise encompassing cell biology, materials engineering, microfluidics, and data analytics. Most Indian biomedical curricula remain limited to two-dimensional culture methods. Recent industry and academic discussions have highlighted significant skill gaps in handling advanced bioprocessing and microphysiological systems technologies, including microfluidic platforms and 3D bioreactor systems, particularly within emerging translational biotechnology ecosystems in India. Furthermore, there is a paucity of trained instructors capable of designing laboratory courses integrating these technologies.

Addressing this gap requires structured national training programs and faculty-exchange models similar to NIH’s Centre for Advancing Translational Sciences (NCATS). Joint certification modules offered by academic consortia and technology providers could standardize competencies.

### 3.2 Infrastructure and equipment accessibility

High-end equipment like bioreactors, perfusion pumps, and high-content imaging systems etc. remain prohibitively expensive for most institutions. Capital expenditure for a modest OOC setup may exceed INR 1.5–2 crore. While Department of science & technology-Sophisticated analytical & technical help institutes (DST- SATHI) program promotes shared infrastructure, dedicated 3D culture facilities are scarce outside metros. Maintenance and import dependencies compound the cost.

Creation of regional 3D culture hubs by department of science & technology (DST), department of biotechnology (DBT), ICMR will allow shared access to capital-intensive platforms. Public-private partnerships (PPP) and subsidized equipment-sharing models can lower entry barriers. Local manufacturing of microfluidic chips, associated consumables, polymer scaffolds, and sensors, leveraging India’s Make-in-India and MedTech missions, can reduce dependency on imports.

### 3.3 Regulatory and policy limitations

At present, CDSCO lacks formal mechanisms to evaluate data generated using 3D culture systems. Without recognized validation standards, pharmaceutical companies hesitate to use these models for regulatory submissions (Piergiovanni et al., 2021; Sewell et al., 2024). The absence of harmonized ethical guidelines for patient-derived organoids or induced-pluripotent-stem-cell (iPSC) lines further constrains clinical translation.

Adopting a two-tier validation framework, viz. one for research-grade 3D models and another for pre-clinical regulatory testing, can provide further clarity. This should be coupled with tax incentives and grant programs for institutions developing validated alternative models. A CDSCO Advisory Committee on Advanced Biomedical Models could mirror the FDA Alternative Methods Program and EMA’s Innovation Task Force (FDA, 2025; EMA, 2026).

### 3.4 Cultural and market barriers

Resistance to change is pervasive in both academia and industry. Traditional reliance on 2D cultures and animal testing is sustained by familiarity, cost, and publication inertia. Moreover, funding agencies often lack defined evaluation criteria for 3D culture projects, resulting in inconsistent peer review (Sewell et al., 2024; Krebs et al., 2025). Private investors perceive these technologies as long-gestation, high-risk ventures.

Mass-awareness initiatives like conferences, demonstration workshops, and dedicated 3D Culture Innovation Challenges, can shift perception. Inclusion of 3D culture metrics in institutional accreditation (e.g., NAAC, NBA) may further incentivize their adoption (NC3Rs, 2026.).

### 3.5 Standardization and reproducibility issues

Reproducibility remains a significant hurdle. Variability in biomaterials, cell sources, and culture media can compromise comparability (Rusyn et al., 2022). Globally, initiatives such as and ISO TC276 provide consensus definitions and reference materials for 3D culture (ISO, 2013). India lacks equivalent standards. Institutions like ICT Mumbai and centre for cellular and molecular biology (CCMB) may lead efforts to draft Indian Standards for 3D Culture Systems (IS-3DCS) in collaboration with various standardization agencies, driving regulatory acceptance. The Bureau of Indian Standards (BIS), the National Standards Body of India, has established a working panel on organoids and organ on chips under the medical equipment and hospital planning division council. This working panel is entrusted with the development of Indian standards and coordinating with similar international standardization activities for organoids and organ on chips.

## 4 Strategic framework for national integration

The transition from isolated research activity to systemic integration demands an enabling framework grounded in four pillars, viz. capacity building, infrastructure development, regulatory enablement, and industry–academia partnerships.

### 4.1 Capacity building and skill development

A National 3D Culture Training Network (N3CTN) could be established under DBT to conduct periodic hands-on workshops across zones. Curricula should include modules on bioreactor design, organ-on-chip fabrication, AI-based image analysis, and regulatory affairs. Integration of 3D culture experiments into postgraduate courses (e.g., M.Tech Bioprocess Technology, Pharmaceutical Biotechnology) would institutionalize awareness. International faculty-exchange programs with NIH, Fraunhofer, and NUS could accelerate capacity building (Vandana et al., 2023).

### 4.2 Enhancing infrastructure and access

Policy mechanisms should prioritize funding of shared 3D culture facilities in academic clusters. However, sustained adoption would require moving from isolated projects to structured programmes. A coherent architecture would include: a national policy coordination layer, regional hubs hosting advanced NAM infrastructure, validation units embedded within hubs, spoke institutions focusing on disease-specific applications and an industry interface for translational deployment. Shared facilities with fee-for-service access can lower entry barriers for MSMEs, prevent duplication, ensure equipment maintenance and quality control and create standardised environments for inter-laboratory benchmarking. This hub-and-spoke approach may balance excellence with accessibility. Incentivizing local fabrication through weighted R&D deductions (under Section 3.5 of the Income Tax Act) would further reduce cost barriers.

### 4.3 Regulatory framework and incentives

To align with FDA and EMA practices, CDSCO may

1. Issue a Guidance Document on 3D Culture and Microphysiological Systems defining validation and qualification criteria for priority CoUs;
2. Establish validation centres under the Indian Pharmacopoeia Commission (IPC) to assess reproducibility;
3. Recognize NAMs data in IND/BE submissions for specific endpoints (e.g., hepatotoxicity, cardiotoxicity); and.
4. Provide special consideration and flexible review schedules for products developed using validated 3D models.

Parallel fiscal incentives such as grant top-ups, GST exemptions, and CSR eligibility etc. may catalyse adoption in private laboratories.

### 4.4 Fostering industry–academia partnerships

**Fig. 2:**
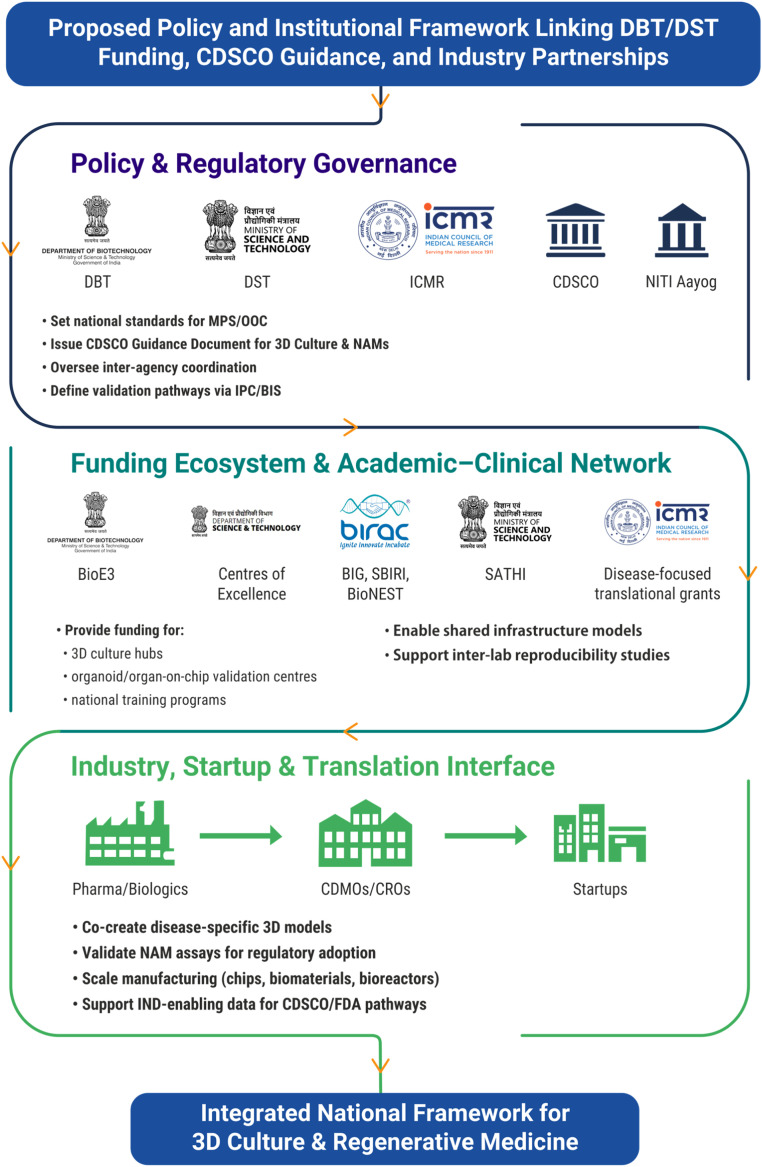
Proposed policy and institutional framework linking DBT/DST funding, CDSCO guidance, and industry partnerships.

3D culture adoption must ultimately translate into industrial relevance. Collaborations between research institutes and pharmaceutical CROs/CDMOs can expedite validation and commercialization. Examples include AstraZeneca–Emulate (Foster et al., 2019) (OOC integration) and Roche’s organoid platforms (Wang et al., 2025). Indian analogues could partner with leading pharmaceutical, biopharmaceutical and vaccine manufacturing companies for disease-model development. Joint IP ownership, milestone funding, and open-access consortia would promote sustainability. Fig. 2 presents the proposed policy and institutional framework linking government funding, CDSCO guidance, and industry partnerships.

## 5 Building awareness and ecosystem engagement

### 5.1 Mass-Scale scientific and policy outreach

For any transformative technology to gain momentum, awareness across researchers, clinicians, regulators, and funding bodies is essential. India’s 3D-culture ecosystem can benefit from a national awareness campaign, similar to the “Human-Relevant Science” initiative launched by the U.S. National Toxicology Program and EMA’s communication strategy for NAMs [(European Medicines Agency EMA, 2026; National Toxicology Program, 2026)].

Flagship platforms such as the Biologics Workshop Series and 3D Cell Culture & Organ-on-Chip (3DCC) Conference at ICT Mumbai have already demonstrated proof-of-concept in mass outreach, drawing hundreds of participants annually from academia and industry. A structured campaign under DBT or DST could brand these efforts as an official National 3D Culture Mission with year-round activities—training weeks, social-media webinars, open days, and translational showcases. A central web portal could disseminate protocols, funding calls, and technology updates, modelled after the NIH NCATS Tissue Chip Consortium. Regular newsletters featuring success stories from India would foster community identity and highlight national capability.

### 5.2 Engaging policymakers and funding agencies

Targeted workshops for DBT, DST, ICMR, and CDSCO officials can demonstrate regulatory relevance and cost-efficiency of 3D models. Policymaker engagement is most effective when supported by pilot case data, such as reduction in animal use or improved assay predictivity.

A formal Policy Roundtable Series on Human-Relevant Biomedical Research, jointly hosted by DBT and CDSCO, may be convened annually to translate scientific progress into updated regulatory and funding norms. Indian funding agencies could adopt a tiered-grant model as follows:

• Tier I: Feasibility and prototype development,
• Tier II: Validation and inter-laboratory reproducibility,
• Tier III: Translation and commercialization readiness.

Integration of such models within BioE3 and ANRF CoE schemes will guarantee continuity and scalability.

### 5.3 Role of media and public engagement

**Fig. 3:**
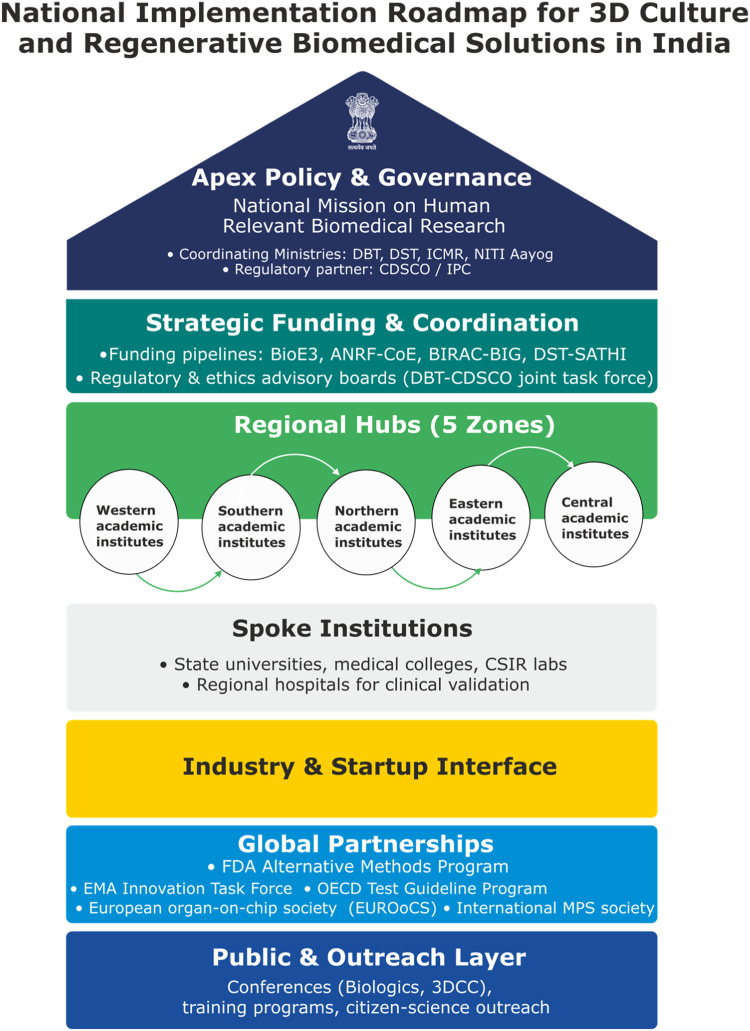
Multi-stakeholder engagement model linking research, regulation, media, and public outreach.

Effective science communication bridges laboratory innovation and public trust. Documentaries, podcasts, and citizen-science modules on the impact of 3D cultures in reducing animal testing can enhance societal acceptance. Partnering with national science channels (DD Science, Vigyan Prasar) and regional language media would amplify reach. Public perception campaigns, like “3D Science for Humane Research”, can position India as a global advocate for ethical biomedicine. Such a multi-stakeholder engagement model linking research, regulation, media, and public outreach has been presented in Fig. 3.

## 6 Integrating regenerative biomedical solutions

### 6.1 Bridging 3D culture with regenerative medicine

3D culture platforms have converged with regenerative medicine through advances in stem-cell biology, biomaterials, and biofabrication. Organoids derived from patient iPSCs are now being evaluated for precision therapy screening, while bioprinted scaffolds aid tissue regeneration (Mirsky et al., 2024; Vandana et al., 2023). Integrating these approaches within Indian biomedical institutions will strengthen translational capacity from bench to bedside.

To operationalize this, India should establish Translational Regenerative Platforms (TRPs)—joint facilities linking academic labs with hospitals. For example, ICT Mumbai could anchor a Skin-and-Wound Regeneration TRP in partnership with tertiary hospitals, validating 3D skin-on-chip systems for diabetic-ulcer therapy. Such frameworks should align with ICMR’s National Guidelines for Stem-Cell Research (2023), ensuring ethical sourcing and informed consent (Department of Biotechnology, 2017).

### 6.2 Clinical translation and industry collaboration

Clinical integration demands co-development between scientists, clinicians, and manufacturers. Hospital-based innovation centres, similar to NHS England’s Biodesign programs, could incubate pilot clinical trials employing organoid or chip-based diagnostics.

Indian contract development and manufacturing organization (CDMOs), like Syngene, OneSource, Sai Life Sciences, Kemwell, Lambda, Anthem Bio can partner with academic groups to validate biomanufacturing processes for cell-based or tissue-engineered products.

Regulatory harmonization with CDSCO is crucial. A dedicated Guidance on Regenerative Products Utilizing 3D Culture Systems could define data requirements for safety, sterility, and functional characterization, mirroring EMA’s Guideline on Human Cell-Based Medicinal Products (EMA/CHMP/410,869/2006 rev 1) (European medicines agency, 2008).

### 6.3 Ethical, legal, and social considerations

While organoid models offer unprecedented personalization, they raise new ethical issues—ownership of patient-derived tissues, genetic privacy, and consent for secondary use (de Jongh et al., 2022). Establishing Ethical Review Boards for Advanced Biomedical Models (ERB-ABM) within institutions can ensure oversight. India’s National Bioethics Committee may extend its mandate to include 3D bioprinting and AI-assisted regenerative research, incorporating FAIR data and open-science principles. Table 2 explains integration of 3D culture platforms with regenerative biomedical applications, based on specific examples, benefits, and policy needs.

**Table 2:** Integration of 3D culture platforms with regenerative biomedical applications: Examples, benefits, and policy needs.

**Application Area**	**Representative 3D Culture / Organ-on-Chip Platform**	**Biomedical / Clinical Objective**	**Key Benefits over Traditional Models**	**Policy and Regulatory Needs (India & Global Alignment)**
Skin and Wound Healing	3D bioprinted skin equivalents; skin-on-chip models (ICT Mumbai, NCCS Pune)	Evaluation of diabetic-ulcer bandages, burn care products, and dermal regeneration	Recreates epidermal–dermal interface; reduces animal dermal toxicity tests	Define validation criteria under CDSCO; align with OECD TG 431 & TG 439; include in ICMR Regenerative Guidelines
Liver Toxicity and Regeneration	Liver organoids and micro-liver-on-chip systems (Wyss Institute, IISc Bangalore)	Predictive hepatotoxicity and metabolic regeneration screening	Maintains hepatic zonation and long-term function	CDSCO acceptance of liver-OOC data for IND toxicology; harmonize with FDA Alternative Methods Program
Cardiac Tissue Repair	iPSC-derived cardiac microtissues and heart-on-chip models, liver-heart 2 organ models (Hesperos), Cardiac chips (Emulate)	Modeling cardiotoxicity and myocardial regeneration	Measures human-specific contractility and ion-channel responses	Develop BIS standards for cardiac OOC validation; align with EMA Advanced Therapy guidelines
Neural Regeneration & Neurodegenerative Disease	Brain organoids and blood–brain-barrier-on-chip platforms (IIT Bombay, CCMB Hyderabad)	Modeling Alzheimer’s and Parkinson’s; testing neuro-protective agents	Mimics neuronal networking and synaptic connectivity	Ethical oversight for iPSC-derived organoids; align with OECD TG 489 for neurotoxicity
Lung and Respiratory Models	Lung-on-chip and alveolar microphysiological systems (ICT Mumbai, IIT Bombay)	Modeling asthma, COPD, and viral infection mechanisms	Dynamic airflow and mechanical strain mimicry	Inter-laboratory validation through DBT–ICMR One-Health framework
Kidney and Renal Disease	Glomerulus-on-chip and kidney organoids (Fraunhofer, NUS)	Nephrotoxicity screening and regenerative nephrology	Real-time filtration metrics and drug response prediction	Include in ANRF translational calls; adapt OECD NAM validation metrics
Cancer and Immunotherapy	Tumoroid and immune-on-chip co-culture systems, Organoplate (Mimetas), immunocompetent tumor models (Dynamic42)	Personalized oncology and immunotherapy testing	Captures tumor microenvironment and immune cell interactions	Create bioethics addendum for patient-derived organoids; enable CDSCO fast-track reviews
Bone and Musculoskeletal Regeneration	Bioceramic-scaffold 3D bone-on-chip constructs, Skeletal muscle module (Hesperos), Bone marrow chips (Emulate), Neuromuscular systems (Raman Lab, MIT)	Screening osteogenic biomaterials and growth factors	Supports biomechanical testing and vascularization studies	Bring under Make-in-India Med-Tech policy; align with ISO 10,993–5 for biocompatibility
Multi-Organ Integration / Systems Biology	Multi-organ or body-on-chip platforms (liver–heart–lung cascade), HUMIMIC (TissUse), 4 organ pumpless system (Hesperos)	Systemic toxicity and pharmacokinetic modeling	Enables human-specific PK/PD prediction and organ crosstalk	Establish CDSCO–FDA collaboration for cross-validation; recognize NAM data in regulatory dossiers

## 7 Roadmap for national-level implementation

### 7.1 Structural framework

A coordinated multi-tier national program may be established as stated in Table 3.

**Table 3:** Multi-tier national program for implementation of NAMs in India.

**Level**	**Function**	**Key Stakeholders**
Policy Apex	National Mission on Human-Relevant Biomedical Research (under DBT)	DBT, DST, ICMR, CDSCO, National institution for transforming india (NITI Aayog)
Regional Hubs (5 zones)	Shared infrastructure for training, validation, and incubation	IITs, ICT, IISc, AIIMS network, and other institutes
Spoke Institutions	Academic partners conducting specific disease-model research	State universities, CSIR labs
Industry Interface	Translation, productization, and scale-up	Biopharma and MedTech companies

Each hub should include dedicated Validation Units accredited by IPC and BIS to benchmark reproducibility and data quality. A Central Data Repository, hosted by BIRAC, can curate validated protocols and inter-lab results, ensuring transparency.

### 7.2 Key performance indicators (KPIs)

To monitor progress, measurable KPIs over a five-year horizon (2025–2030) are suggested as follows:

1. Capacity Building: ≥ 2000 researchers trained nationwide.
2. Infrastructure: ≥ 10 regional 3D culture hubs operational.
3. Research Output: ≥ 500 peer-reviewed publications + 50 patents.
4. Regulatory Impact: Inclusion of ≥ 5 validated 3D models in CDSCO review.
5. Industry Adoption: ≥ 20 pharma/biotech collaborations leveraging 3D platforms.
6. Animal Use Reduction: ≥ 30% decline in animal-based pre-clinical studies in publicly funded projects.

These metrics can be integrated into DBT’s performance dashboards and evaluated annually through independent review.

### 7.3 Funding and sustainability

Creating a nationally scalable ecosystem for 3D culture, organoid science, and regenerative biomedical technologies in India requires a diversified, multi-agency funding architecture rather than isolated programmatic support. Several Government of India agencies have already introduced forward-looking schemes that can be strategically aligned to build a sustainable national framework.

At the forefront, the Department of Biotechnology’s flagship BioE3 Biomanufacturing and Biofoundries Initiative provides a dedicated platform for enabling advanced biomanufacturing capabilities and translational bioengineering research (Department of Biotechnology, 2026). Complementing this, BIRAC’s established programs—Biotechnology Ignition Grant (BIG), small business innovation research initiative (SBIRI), and Biotechnology Industry Partnership Programme (BIPP)—serve as India’s most effective early-stage innovation vehicles, supporting ideation, prototype development, and commercialization-readiness for deep-tech biotech ventures (BIRAC, 2026; BIRAC, 2026; BIRAC, 2026). Additionally, the DBT scientific infrastructure access for harnessing academia-university research joint collaboration (SAHAJ) Scheme, which facilitates seamless access to high-end research infrastructure, can directly support national-level facilities for organoid, organ-on-chip, and microfluidic testing (DBT, 2026).

In parallel, the Department of Science and Technology’s sophisticated analytical & technical help institutes (SATHI) Program offers a proven model for shared scientific infrastructure, built on sustainable fee-based access, long-term equipment maintenance strategies, and multi-institutional participation (Department Of Science & Technology, 2026.). These centres are ideally positioned to host regional 3D culture hubs, reducing capital burden on Tier-II and Tier-III institutions and ensuring broader accessibility.

Clinical translation and validation capabilities can be integrated through the ICMR Centres for Advanced Research (CAR) and the ICMR First-in-the-World Challenge, which enable disease-focused, clinician-driven translational projects—an essential requirement for validating organoid and MPS platforms for clinical or regulatory use (ICMR, 2026; ICMR, 2025). Meanwhile, the ANRF provides a structural mechanism to build convergent Centres of Excellence that unify biological sciences, materials engineering, clinical research, AI, and regulatory science (ANRF, 2026).

Because 3D culture and microphysiological systems depend heavily on advanced sensors, microfabrication, imaging systems, and computational pipelines, technology missions outside the traditional biosciences are also critical. The Ministry of Electronics and Information Technology (MeitY), through the India AI Mission, the PLI Scheme for Electronics Manufacturing, and the Design-Linked Incentive (DLI) program, supports indigenous development of semiconductors, microfluidics, embedded sensing, and AI-driven analytical tools all essential for enabling scalable organ-on-chip and 3D biomanufacturing platforms (MeitY, 2026; Industrial Finance Corporation of India, 2026; ISM, 2026).

Furthermore, the Research, Development and Innovation (RDI) Scheme, introduced by department for promotion of industry and internal trade (DPIIT) to catalyse private-sector R&D investment, can accelerate co-funded development of indigenous bioprocessing equipment, imaging systems, organoid culture consumables, and microfluidic chip manufacturing (DPIIT, 2026; Department of Science & Technology, 2026). This aligns directly with India’s strategic objective of reducing import dependency in critical life-science instrumentation.

Sectoral agencies such as defence research and development organization (DRDO) and board of research in nuclear sciences (BRNS) (DAE) can also support specialised validation studies where organoid and microphysiological systems are uniquely suited—for example, defence toxicology, aerospace physiology, radiobiology, and radiation countermeasure testing. Their mission-driven funding structures create new translational opportunities for expanding 3D culture technologies beyond traditional biomedical domains.

To ensure long-term sustainability, India must adopt a hybrid financing model that combines central grants, competitive translational funding, industry co-investment through the RDI scheme, PPP-based infrastructure management, philanthropic support, and a SATHI-style fee-for-access model. International best practices indicate that diversified portfolios and public–private partnerships significantly enhance research infrastructure sustainability and innovation output (Araújo and Sutherland, 2010). Integrating these approaches will allow India to move from isolated, grant-dependent research pockets to a national, mission-driven ecosystem capable of sustaining large-scale deployment of 3D culture and regenerative biomedical technologies.

### 7.4 International partnerships

**Fig. 4:**
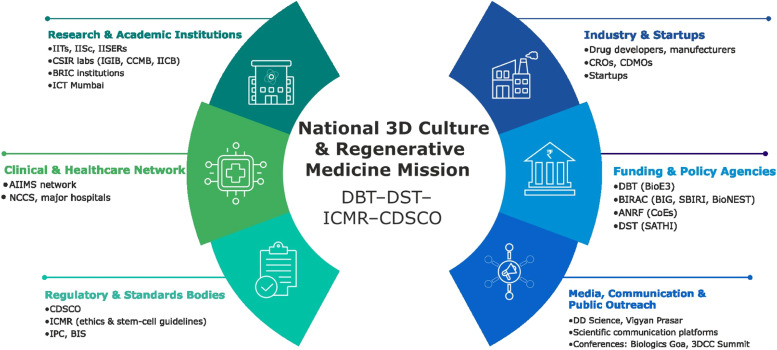
National implementation roadmap: policy, infrastructure, funding, and international integration. The figure visualizes a multi-tier national ecosystem connecting policy, infrastructure, research, and industry under a unified mission.

Global partnerships will accelerate India’s learning curve. MoUs with the FDA Alternative Methods Working Group, EMA Innovation Task Force, and OECD Test Guidelines Program can formalize knowledge exchange. Participation in international validation studies (e.g., Inter-agency MPS Consortium) will benchmark Indian data globally, ensuring mutual recognition. Reciprocal training fellowships for Indian scientists at Wyss Institute, Fraunhofer, and NUS Centre for Life Sciences will nurture global expertise. Fig. 4 depicts the sequential and inter-linked flow of governance, funding, research execution, and translation pathways—each anchored by existing or proposed national programs.

## 8 Conclusion and policy outlook

The global transition toward human-relevant, ethical, and predictive biomedical systems is no longer optional—it is a structural shift redefining how therapies are discovered, evaluated, and manufactured. For India, the adoption of 3D culture, organoids, microphysiological systems, and regenerative biomedical platforms represents far more than a technological upgrade. It is a strategic national imperative that directly influences India’s scientific sovereignty, pharmaceutical leadership, public health preparedness, and global competitiveness. By integrating these platforms into mainstream biomedical R&D, India can reduce its dependence on imported technologies, accelerate translational timelines, and move toward ethical, patient-specific test systems that meet evolving global regulatory expectations from the FDA, EMA, and OECD. Equally important, such systems can drive affordable innovation, enabling India to offer the world cost-effective alternatives for drug development, toxicity assessment, personalised medicine, and regenerative therapies.

Considering the significant resources needed for the ambitious deployment plan and considering its dependence on a stable supply chain, India could prioritize the validation of a few in-house, high-impact, CoU-driven MPS platforms, particularly for liver toxicity, kidney toxicity, liver–kidney interactions, hepatic metabolism, cardiotoxicity, vaccine safety, and biologics assessment, with support of shared national infrastructure and early regulatory engagement. Beyond achieving national self-reliance in drug discovery and public health, India has a unique opportunity to contribute to the global MPS ecosystem based on its strong pharmaceutical and biopharmaceutical sectors, diverse patient populations, engineering and microfabrication capabilities, and capability of facilitating cost-effective innovations. Thus, India can play a leadership role in developing affordable and scalable MPS technologies, generating Indian population-relevant datasets, deploying these models for development of biosimilars, complex generics, peptides, vaccines, and advanced therapies, and serving as a global hub for affordable training, capacity building, and regulatory science. By actively participating in international validation studies, OECD-aligned frameworks, and global regulatory collaborations, India can contribute not only to the adoption of human-relevant NAMs but also to the establishment of globally accepted standards for next-generation risk assessment and drug development.

Realizing this vision requires harmonized national standards through CDSCO, IPC, and BIS; skilled workforce development through DBT, DST, ICMR, MeitY, and academic institutions; and deep industry integration through PPP models, the RDI scheme for private R&D investment, and sector-specific missions. With DBT’s BioE3 biomanufacturing hubs, DST-SATHI shared facilities, ICMR Centres for Advanced Research, BIRAC’s BIG/SBIRI/BIPP accelerators, ANRF Centres of Excellence, MeitY’s AI and electronics missions, DRDO and BRNS translational needs, and state-supported biotech clusters, India already possesses the institutional scaffolding needed to scale these technologies nationally.

A coordinated National Mission on 3D Culture, Organoid Science & Regenerative Biomedical Innovation, jointly anchored by DBT, DST, ICMR, CDSCO, MeitY, DAE/BRNS, DRDO, ANRF, and BIRAC, and formally aligned with India Vision 2047, will unify currently fragmented efforts across research, regulation, manufacturing, defence, and clinical translation. Such a Mission can serve as the apex driver for scientific validation, regulatory convergence, infrastructure creation, indigenous instrumentation, AI-driven analytics, and national-scale adoption.

The immediate next step is the formulation of a National Policy White Paper, jointly authored by DBT, DST, ICMR, MeitY, CDSCO, DRDO, BRNS, and ANRF, outlining regulatory pathways, funding mechanisms, institutional mandates, validation networks, ethical guidelines, and a five-year implementation roadmap. Releasing this by 2026 would strategically position India to lead the Global South in ethical, scalable, and human-relevant biomedical innovation, setting the foundation for a research ecosystem that is humane, sustainable, self-reliant, and globally credible.

**Fig. 5:**
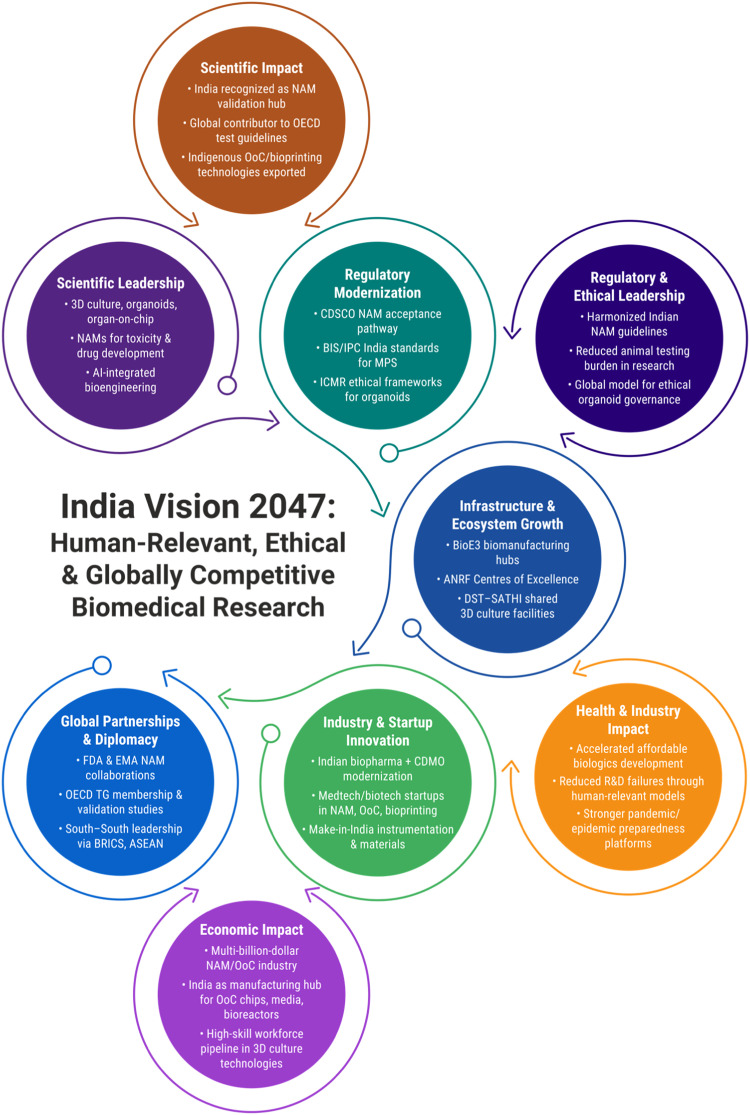
Vision 2047 alignment: India as a Global South leader in ethical, human-relevant biomedical research.

Fig. 5 depicts India’s vision in near future as a global south leader in ethical, human-relevant biomedical research.

## CRediT authorship contribution statement

**Prajakta Dandekar:** Writing – review & editing, Writing – original draft, Visualization, Supervision, Methodology, Investigation, Conceptualization. **Ratnesh Jain:** Writing – review & editing, Writing – original draft, Visualization, Methodology, Investigation, Conceptualization. **Surat Parvatam:** Writing – review & editing, Writing – original draft, Visualization, Project administration, Methodology, Investigation, Conceptualization. **Viraj Mehta:** Writing – review & editing, Writing – original draft, Visualization.

## Declaration of competing interest

The authors declare that they have no known competing financial interests or personal relationships that could have appeared to influence the work reported in this paper.

## Data Availability

No data was used for the research described in the article.

## Appendix Supplementary materials

Image, application 1
