# Supplementary material for: Strategic integration of 3D Cell culture and regenerative biomedical technologies in India’s biomedical ecosystem: Aligning with the global transition toward human-relevant drug development
Source: NAM J. 2026 Jul 16;2:100116. doi: 10.1016/j.namjnl.2026.100116 (PMC13393737; doi:10.1016/j.namjnl.2026.100116)
Supplement: Supplementary file 1 [file mmc1.docx]

**Strategic Integration of 3D Cell Culture and Regenerative Biomedical Technologies in India’s Biomedical Ecosystem: Aligning with the Global Transition Toward Human-Relevant Drug Development**

Prajakta Dandekar *^a$^ Ratnesh Jain*^b^ Surat Parvatam^c^  Viraj Mehta^d^

^a^ Department of Pharmaceutical Sciences and Technology, Institute of Chemical Technology, Mumbai, India

^b^ Department of Biological Sciences and Biotechnology, Institute of Chemical Technology, Mumbai, India

^c^ Program Director, Humane World for Animals India, Hyderabad, India

^d^ Associate Research Scientist, Sai Life Sciences, Hyderabad, India

*Joint authors

^$^Corresponding author’s email address: pd.jain@ictmumbai.edu.in

Table S1. Examples of OECD guidelines that validated NAMs for safety assessment

| **Test name** | **In vitro/in chemico/ in silico method** | **OECD Guideline No.** |
| --- | --- | --- |
| Skin irritation/corrosion | **In vitro** skin corrosion: reconstructed human epidermis (RHE) test method  **In vitro** skin irritation: reconstructed human epidermis test method  **In Vitro** 3T3 NRU Phototoxicity Test  **Combinatorial** Integrated Approach on Testing and Assessment (IATA) for Skin Corrosion and Irritation | OECD TG 431  OECD TG 439      OECD TG 432  OECD GD 203 |
| Eye irritation | **In vitro** Short Time Exposure In Vitro Test Method (STE)​    **In vitro** Reconstructed human Cornea-like Epithelium (RhCE) test method​    **In vitro** Vitrigel-Eye Irritancy test Method ​    **In vitro** In vitro Macromolecular Test Method ​    **In vitro** Reconstructed Human Cornea-like Epithelium Test Method Time-to-Toxicity (HCE-TTT)​    **Combinatorial** Integrated Approaches to Testing and Assessment (IATA) for Serious Eye Damage and Eye Irritation​  (animals as a last resort)    **Combinatorial** Defined Approaches for Serious Eye Damage and Eye Irritation | OECD TG 491      OECD TG 492      OECD TG 494    OECD TG 496      OECD TG 492B        OECD GD 263          OECD TG 467 |
| Skin sensitization | **In chemico** Direct Peptide Reactivity Assay (DPRA) ; Amino Acid Derivative Reactivity Assay (ADRA); Kinetic Direct Peptide Reactivity Assay (kDPRA).​    **In chemico + gene expression analysis** ARE-Nrf2 luciferase KeratinoSens™ test method; ARE-Nrf2 luciferase LuSens test method​; Epidermal sensitization Assay – EpiSensA    **In vitro** Human Cell Line Activation test (h-CLAT) ; U937 cell line activation Test (U-SENS™); Interleukin-8 Reporter Gene Assay (IL-8 Luc assay); Genomic Allergen Rapid Detection (GARD™) for assessment of skin sensitisers (GARD™skin).    **Combinatorial** Defined Approaches on Skin Sensitisation ​ | OECD 442C          OECD 442D            OECD 442E              OECD DA 497 |
| Short-term fish toxicity | **In vitro:** Replacement/reduction  Fish Embryo Acute Toxicity, FET      Fish Cell Line Acute Toxicity: The RTgill-W1 Cell Line Assay | OECD Test Guideline 236    OECD Test Guideline 249 |
| Genotoxicity assessment | **In vitro** Bacterial Reverse Mutation Test  **In vitro** chromosomal aberration test  **In Vitro** Mammalian Cell Gene Mutation Tests  **In Vitro** Mammalian Cell Micronucleus Test  **In Vitro** Mammalian Cell Gene Mutation Tests Using the Thymidine Kinase Gene | OECD TG 471  OECD TG 473  OECD TG 476  OECD TG 487  OECD TG 490 |
| Endocrine disruption | Performance‑Based Test Guideline for Stably Transfected Transactivation **In Vitro** Assays to Detect Estrogen Receptor Agonists and Antagonists  **In vitro** H295R Steroidogenesis Assay  **In vitro** Stably Transfected Human Androgen Receptor Transcriptional Activation Assay for Detection of Androgenic Agonist and Antagonist Activity of Chemicals  Performance‑Based Test Guideline for Human Recombinant Estrogen Receptor (hrER) **In Vitro** Assays to Detect Chemicals with ER Binding Affinity | OECD TG 455  OECD TG 456  OECD TG 458  OECD TG 493 |
